# Non-A Hepatitis B Virus Genotypes in Antenatal Clinics, United Kingdom

**DOI:** 10.3201/eid1311.070578

**Published:** 2007-11

**Authors:** Samir Dervisevic, Samreen Ijaz, Shahneila Chaudry, Richard S. Tedder

**Affiliations:** * University College London Hospitals National Health Service Foundation Trust, London, United Kingdom; † Health Protection Agency, London, United Kingdom

**Keywords:** Hepatitis B virus, hepatitis B virus DNA, hepatitis B virus e antigen, antibody to hepatitis B virus e protein, precore mutations, basal core promoter, HBV genotypes, pregnancy, research

## Abstract

In the United Kingdom, the National Screening Programme for identification of hepatitits B virus (HBV) infection in pregnant women uses HBV e antigen (HBeAg) and antibody to HBeAg (anti-HBe) as markers of infectivity to determine use of immunoglobulin for hepatitis B. Serum samples from 114 HBV-infected women were analyzed. Viral loads correlated with HBeAg/anti-HBe status and viral genotypes. Among 95 mothers whose serum contained anti-HBe, viral loads ranged between undetectable and 8.6 × 10^6^ IU/mL (median 228 IU/mL). Ten (10.5%) of these mothers had plasma viral loads >10^4^ IU/mL; 6 were infected with genotype E and one each with genotypes A, B, C, and D. All viruses had precore stop codon or basal core promoter mutations. Preponderance of genotypes other than A among antenatal mothers in the United Kingdom reflects increasing globalization and trends in immigration. HBeAg serostatus is no longer sufficiently accurate for inferring potential infectivity of pregnant HBV carriers.

## Abstract 2

Serostatus for viral e antigen is no longer accurate for inferring potential infectivity of pregnant virus carriers.

Hepatitis B virus (HBV) infection remains a major health problem worldwide and mother-to-infant transmission represents one of the most efficient ways of maintaining hepatitis B carriage in any population. Intervention to prevent this route of infection is a key part of the global program of hepatitis B control. Although there are 3 routes of transmission of HBV from infected mothers to their infants, including transplacental and postnatal, most transmission is likely to occur perinatally at the time of labor and delivery (*1*). HBV e antigen (HBeAg) in maternal serum is associated with high infectivity; in the absence of intervention after delivery, including both passive and active immunization, 90% of babies born to carrier mothers whose serum contains HBeAg will become chronically infected with HBV (*2*,*3*). Babies born to mothers whose serum contains antibody to HBeAg (anti-HBe) become infected far less frequently (*4*). However, babies who are infected may be at risk of developing fulminant hepatitis B (*2*).

The prevalence of HBV infection in the United Kingdom is low (0.4%) (*5*). In the late 1990s, the World Health Organization (WHO) recommended introduction of global universal hepatitis B immunization programs (*6*); by March 2002, a total of 151 countries, including 34 in Europe, had introduced HBV vaccine within their national immunization programs. However, current control of mother-to-infant HBV transmission in the United Kingdom is based on selective hepatitis B immunization of infants at risk. A recent WHO survey in Europe indicated that 8 other countries also used this approach (*7*). This requires routine antenatal screening for HBV infection (*8*,*9*), offered by 34 countries in Europe, with infants born to all hepatitis B–infected mothers being offered immediate postnatal active immunization with hepatitis B vaccine. In the United Kingdom, babies at highest risk for infection, those born to mothers whose serum does not contain anti-HBe, are offered additional passive immunization prophylaxis (*10*) with 200 IU of hepatitis B immunoglobulin (HBIg) within 24 hours of delivery. In this protocol, detection of anti-HBe is used to infer low infectivity.

Despite full prophylaxis for neonates, a small proportion of infants still become persistently infected (*11*–*13*) and are at risk of developing sequelae of chronic HBV infection and increasing the HBV reservoir. Although the causes for these failures could be many, we noted that in management of HBV–infected healthcare workers, inference of infectivity is now based upon plasma viral load for HBV rather than HBe markers. Until 2001 in the United Kingdom, fitness of an HBV-infected healthcare worker to undertake invasive procedures was predicated upon absence of HBeAg, a protocol that was found to enable transmission to patients (*14*). All transmission involved infections by viruses with the pre-core premature stop codons, which reflected changes in viral genotypes caused by increased migration in UK healthcare workers. To investigate potential inappropriate categorization of infection risk through continued use of HBe markers in the antenatal setting, we undertook a study to relate HBe markers to HBV DNA levels and genotypes as predictors of potential infectivity.

## Patients and Methods

### Patients

As part of routine antenatal care, screening for HBV infection is offered to all pregnant mothers at the University College London Hospital. Pregnant HBV carriers who came to the hospital from September 1989 through September 2004 were identified. Serum samples from 114 HBV-infected mothers were available for further testing. Ethnic origin of mothers was not recorded.

### Serologic Tests

Serum was separated and stored at –20°C in the Department of Virology, University College London Hospital, in accordance with laboratory policy to archive samples from carriers because of the long incubation time to clinical expression of HBV-related chronic liver disease. Samples would have been tested at initial collection for HBsAg by using a range of commercial assays and had reactivity confirmed by neutralization tests. Further testing for HBeAg, anti-HBe, antibody to hepatitis B virus core antigen (anti-HBc), and immunoglobulin M to HBc would have been performed routinely to determine the need for HBIg and confirm carrier status.

### Quantitative PCR and Sequencing

Viral load for HBV DNA was measured as described (*15*). Briefly, HBV DNA was extracted from serum by using the QIAamp Virus BioRobot 9604 and QIAamp96 Virus Kit reagents (QIAGEN, Hilden, Germany) in accordance with the manufacturer’s instructions. Twenty microliters of extract was used for input into a Taqman-based assay for HBV DNA in an ABI Prism 7000 sequence detection system (Applied Biosystems, Foster City, CA, USA). Serum samples containing >100 IU/mL of viral DNA were selected for sequencing. Five microliters of extract was used for nested amplification of the entire virus surface antigen gene as described (*16*). We amplified precore and basal core promoter (BCP) regions of HBV DNA from anti-HBe–positive serum samples that contained >10^4^ IU/mL of HBV DNA. Briefly, 5 μL of extracted HBV DNA was amplified by using primers H4072, 5′-TCTTGCCCAAGGTCTTACAT-3′, and C outer (outer antisense), 5′-TCCCACCTTATGAGTCCAAG-3′, in the first round and primers H4072 (primer sequence as above) and C inner, 5′-CAGCGAGGCGAGGGAGTTCTTCTT-3′, in the second round. Conditions for amplification were the same for both rounds: 94°C for 4 min; 35 cycles at 94°C for 30 s, 55°C for 30 s, and 72°C for 1 min; and a final extension at 72°C for 5 min. Amplicons were sequenced with CEQ 8000 Genetic Analysis Systems (Beckman Coulter, Fullerton, CA, USA) in accordance with the manufacturer’s instructions.

Generated nucleotide sequences were assembled and analyzed by using the SeqMan program (DNASTAR Inc., Madison, WI, USA). Alignments of nucleotide sequences were conducted to determine phylogenetic relationships between different isolates of HBV by using the MegAlign program (DNASTAR, Inc.). Data were used to construct a phylogenetic tree. Further analysis was also conducted with HBV STAR analysis, which assigns HBV genotypes by using a position-specific scoring matrix (www.vgb.ucl.ac.uk/star.shtml). Statistical significance was determined by using the Fisher exact test in the Arcus Quickstat package (www.camcode.com/arcus.htm).

## Results

Thirteen (11.4%) of 114 HBsAg-positive serum samples contained detectable HBeAg, 95 (83.3%) contained anti-HBe, and 6 (5.3%) did not contain HBeAg or anti-HBe. HBIg had been recommended only for babies born to 13 mothers whose serum contained HBeAg and to 6 mothers whose serum did not contain HBeAg or anti-HBe.

### HBV DNA, HBeAg, Anti-HBe, and Genotypes

HBV DNA was detected in 96 (84%) serum samples (Figure). The 13 HBeAg-positive serum samples had viral loads that ranged from 7.8 × 10^5^ to 1 × 10^8^ IU/mL (median 2.2 × 10^7^ IU/mL). In 95 samples positive for anti-HBe, viral loads ranged from undetectable to 8.6 × 10^6^ IU/mL (median 228 IU/mL). In 6 serum samples with neither e markers detected, viral loads ranged from undetectable to 750 IU/mL (median 120 IU/mL). Ten (10.5%) of 95 anti-HBe–positive samples had viral loads >10^4^ IU/mL, ranging up to 8.6 × 10^6^ IU/mL.

**Figure:**
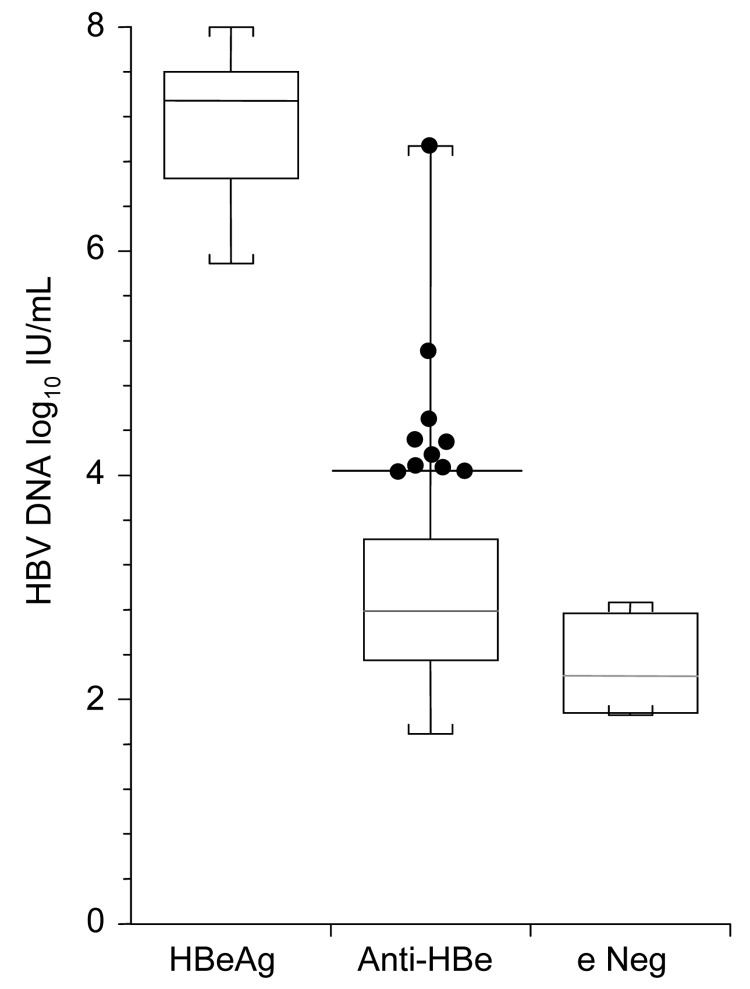
Box and whisker plots of hepatitis B virus (HBV) load in 3 groups of mothers whose serum contained hepatitis B virus e antigen (HBeAg), antibody to hepatitis B virus e antigen (anti-HBe), or neither of these markers (e Neg). Boxes are middle quartiles, horizontal lines are medians, whiskers are ranges, and dots represent 10 anti-HBe–seropositive mothers whose serum contained >10^4^ IU/mL HBV DNA. Thirty-three anti-HBe–seropositive mothers and 1 mother whose serum did not contain either marker did not have detectable HBV DNA (<50 IU/mL).

HBV DNA extracts from 40 serum samples were successfully sequenced and genotyped. Genotypes E (13/40, 32.5%) and B (10/40, 25%) predominated. Genotypes A (6/40, 15%), C (9/40, 22.5%), and D (2/40, 5%) accounted for the remaining genotypes. In 10 serum samples with viral loads >10^4^ IU/mL and anti-HBe, the distribution of genotypes was significantly different, with an excess of genotype E (p = 0.05 by Fisher exact test; Table). Of the 10 viruses infecting these mothers, 5 had precore changes, 3 had precore and BCP changes, and 2 had BCP changes.

**Table:** Characteristics of HBV in 10 infected mothers seropositive for antibodies to HBV e antigen and with serum HBV DNA levels >10^4^ IU/mL*

Carrier mother	Genotype	HBV DNA, IU/mL	Basal core promoter	Precore†
1	D	2.5 × 10^4^	W	W28
2	E	1 × 10^4^	W	W28
3	E	1.1 × 10^4^	130K/131I	W28 G29D
4	C	1.1 × 10^5^	130K/131I	W
5	B	1.7 × 10^4^	130K/131I	W28
6	E	8.6 × 10^6^	W	W28
7	A	3.4 × 10^4^	130K/131I	W
8	E	1.9 × 10^4^	W	W28
9	E	2.2 × 10^4^	130K/131I	W28
10	E	1.0 × 10^4^	W	W28

*HBV, hepatitis B virus; W, wild-type sequence. †W28, premature stop at codon 28.

## Discussion

This study investigated the continuing use in the United Kingdom of maternal HBeAg markers as predicators for enhanced neonatal HBIg prophylaxis in addition to neonatal vaccine. Among 51 countries in Europe, the United Kingdom, along with 14 others, has elected to not introduce routine neonatal HBV immunization at this time (*7*), rather opting for selective screening in the antenatal clinics and targeted prophylaxis to infants born to infected mothers. This policy requires efficient HBV screening in clinics. We recognize that resources required for implementing this policy are not available in many countries. This policy has the advantage of enabling the addition of HBIg to prophylaxis for infants born to mothers with high infectivity, although how widespread this practice is in Europe is not known. HBIg is a costly intervention and is limited by availability. It is also a blood product that has the risk for transmission of prion disease through inclusion of donations from persons with variant Creutzfeldt-Jakob disease in the plasma pool.

Serum samples from 114 hepatitis B carrier mothers were examined. Thirteen (11.4%) contained HBeAg, with concentrations of HBV DNA ranging from 7.8 × 10^5^ to 1 × 10^8^ IU/mL. All infants born to these mothers would have been at high risk of acquiring HBV and should have been offered active immunization with the HBV vaccine, as well as passive prophylaxis with HBIg. Six serum samples did not contain detectable HBeAg or anti-HBe. Although HBV DNA levels were low in all samples, infants of these mothers would still have been given HBIg in accordance with guidelines, probably unnecessarily. Eighty-five of 95 serum samples with anti-HBe had HBV DNA levels <10^4^ IU/mL and infants of these mothers would have received only active immunization. Ten (10.5%) of 95 serum samples had HBV DNA concentrations >10^4^ IU/mL, and 2 (2.1%) of these had high viral loads >10^5^ IU/mL (110,000 IU/mL and 8,690,000 IU/mL, respectively). The infants of these mothers would not have been offered HBIg on the basis of maternal anti-HBe as a marker of low infectivity. It is not known whether such infants are more likely to become infected as they had received only vaccine prophylaxis.

In the late 1970s in Japan, use of anti-HBe as a marker for low infectivity had been based on the observation (*17*) that anti-HBe–seropositive carriers were unlikely to transmit hepatitis B sexually or to their infants. This belief was verified by observations in genitourinary medicine clinics (*18*) and included in Department of Health policy in the United Kingdom that allowed hepatitis B carriers to conduct exposure-prone procedures if their serum did not contain HBeAg. In retrospect, it seems likely that at the time of promulgation of these guidelines, most infections with hepatitis B virus in the UK workforce would have been with genotype A. This Department of Health policy continued until description of several surgical transmissions from HBV-infected healthcare workers (*14*) and the recognition that some carriers whose serum contained anti-HBe had high viral loads. After this episode, estimation of plasma HBV DNA load was introduced to manage infected healthcare workers (*19*). Most of the surgeons involved had been born in HBV-endemic countries outside Europe and would have been infected by a genotype other than genotype A. All viruses transmitted had premature stop codons in the precore region, which are changes not commonly seen in genotype A infections.

Dominance of nongenotype A infections among antenatal women in the United Kingdom, with genotype A accounting for only 15%, is explained by the recent observation that a net of ≈6,000 HBV carriers immigrate annually to the United Kingdom (*5*) from areas such as eastern Europe, where non-A viruses predominate. This immigration will undoubtedly change clinical expression of HBV carriage in the United Kingdom and provides an example of reemergence of an old virus disease with different characteristics. Flaring (increase in alanine aminotransferase levels caused by immune-mediated destruction of hepatocytes) and late escape (elevated levels of viral DNA) of virus from host-dependent modulation (innate or adaptive immune responses to infection with HBV) is seen more frequently with non-A viruses than with European genotype A HBV. All but 1 of the viruses in serum samples from 10 anti-HBe carrier mothers who had high viral loads were non-A, and all carried changes associated with enhanced virus replication. Five had changes in the precore region, 2 had changes in the BCP, and 3 had changes in both regions.

BCP mutations at nucleotide positions 1762/1764 and precore mutation G1896A, which results in a premature stop at codon 28, reduce or prevent expression of HBeAg. Both mutations are likely the result of virus evolution and selection of the fittest strains (*20*) during host immune responses. BCP changes result in decreased transcription of precore/core mRNA, reduced secretion of HBeAg (*21*), and enhanced virus production in vitro (*22*,*23*). These changes have been detected more often in viruses with genotypes A and C than in those with genotypes B, D, and E (*24*). However, in our study, BCP mutations were seen in viruses with genotypes A, C, D, and E. These mutations are thought to arise before precore changes (*25*). The premature stop precore mutation is restricted to HBV genotypes containing a thymidine at nucleotide position 1858, which is required for stabilizing the stem loop structure (*26*). This mutation, which is found in viruses with genotypes B, D, E, G and some strains with genotypes C and A (*27*), explains the high prevalence of premature stop variants in Asia and the Mediterranean region, where predominant genotypes are B, C, and D and their previous low prevalence in the United Kingdom. Our study demonstrates changing phenotypes of virus infections caused by population movement. These changes are unlikely to be limited to the United Kingdom and have wider implications for infectious diseases globally.

Our study demonstrates that reliance on only HBV serologic markers leads to misclassification of HBV carrier mothers. A proportion of low-infectivity carriers had high levels of virus in plasma but their infants would not have received optimal enhanced prophylaxis with postnatal HBIg. This policy could allow avoidable breakthrough infections in infants. In view of the influx of immigrant HBV carriers into the United Kingdom, a new HBV antenatal screening strategy is needed to identify and offer adequate protection to infants at risk of acquiring HBV infection. Quantification of HBV DNA is a more objective direct measure of potential infectivity and brings this procedure in line with management of HBV-infected healthcare workers (*19*). However, the cut-off level of HBV DNA needed to define potential activity has yet to be established. Finally, given the emerging pattern of an overall increase in HBV carriage in the United Kingdom, consideration should once again be given to a national program of immunization of infants.
